# Soluble adenylyl cyclase mediates hydrogen peroxide-induced changes in epithelial barrier function

**DOI:** 10.1186/s12931-016-0329-4

**Published:** 2016-02-08

**Authors:** Pedro Ivonnet, Hoshang Unwalla, Matthias Salathe, Gregory E. Conner

**Affiliations:** Division of Pulmonary, Allergy, Critical Care and Sleep Medicine, Miller School of Medicine, University of Miami, 1600 NW 10th Ave, Miami, 33136 FL USA; Department of Immunology, Herbert Wertheim College of Medicine, Florida International University, Miami, FL 33199 USA; Department of Cell Biology, Miller School of Medicine, University of Miami, 1600 NW 10th Ave, Miami, 33136 FL USA

**Keywords:** Soluble adenylyl cyclase, Hydrogen peroxide, Airway epithelium, EP1

## Abstract

**Background:**

Elevated H_2_O_2_ levels are associated with inflammatory diseases and H_2_O_2_ exposure is known to disrupt epithelial barrier function, leading to increased permeability and decreased electrical resistance. In normal human bronchial epithelial (NHBE) cells, fully differentiated at the air liquid interface (ALI), H_2_O_2_ activates an autocrine prostaglandin pathway that stimulates transmembrane adenylyl cyclase (tmAC) as well as soluble adenylyl cyclase (sAC), but the role of this autocrine pathway in H_2_O_2_-mediated barrier disruption is not entirely clear.

**Methods:**

To further characterize the mechanism of H_2_O_2_-induced barrier disruption, NHBE cultures were treated with H_2_O_2_ and evaluated for changes in transepithelial resistance and mannitol permeability using agonist and inhibitors to dissect the pathway.

**Results:**

A short (<10 min) H_2_O_2_ treatment was sufficient to induce resistance and permeability changes that occurred 40 min to 1 h later and the changes were partially sensitive to EP1 but not EP4 receptor antagonists. EP1 receptors were localized to the apical compartment of NHBE. Resistance and permeability changes were sensitive to inhibition of sAC but not tmAC and were partially blocked by PKA inhibition. Pretreatment with a PLC inhibitor or an IP3 receptor antagonist reduced changes in resistance and permeability suggesting activation of sAC occurred through increased intracellular calcium.

**Conclusion:**

The data support an important role for prostaglandin activation of sAC and PKA in H_2_O_2_-induced barrier disruption.

**Electronic supplementary material:**

The online version of this article (doi:10.1186/s12931-016-0329-4) contains supplementary material, which is available to authorized users.

## Background

Junctional complexes are composed of an assortment of proteins that anchor cells to each other and their basement membranes, thereby forming a stable tissue that serves to regulate passage of materials across the mucosa. Regulation of the apical junctional complex is key to epithelial barrier function. Numerous studies have shown changes in transepithelial permeability and electrical resistance can occur rapidly and reversibly and mirror changes in intercellular junction structure. Loss of barrier function is often associated with inflammation [1]. A large number of studies have shown that H_2_O_2_, frequently elevated in inflammatory diseases, reversibly alters paracellular epithelial permeability and resistance (e.g., [2–4]). H_2_O_2_ on epithelial surfaces can result from the respiratory burst of invading phagocytes or from epithelial cells themselves that produce H_2_O_2_ through the enzymatic action of the NADPH oxidases Duox 1 & 2 [5–7].

The mechanism by which H_2_O_2_ alters permeability and transepithelial resistance is multifactorial and differs between differentiated epithelia, endothelia and cell lines (e.g., [8]), but uniformly entails junctional protein re-distribution (e.g., [2, 9–13]). Occludin, ZO1 and claudins are released from junctions after H_2_O_2_ exposure. H_2_O_2_ alteration of the epithelial barrier is known to rely on increased protein tyrosine phosphorylation by inhibition of protein tyrosine phosphatase [8, 11], p38 MAP kinase activity [14] and dephosphorylation of occludin by PP2A in a Src kinase-dependent fashion [15]. Involvement of protein kinase C has been reported in some cases [16] but ruled out in others [3].

Studies in bovine tracheal epithelia [17], in human airway epithelial cell lines [18, 19] and more recently in fully differentiated normal human bronchial epithelial (NHBE) cells [20] show that acute exposure to H_2_O_2_ stimulates an autocrine prostanoid signaling pathway that elicits an increase in CFTR-mediated anion secretion, which can be seen in Ussing chamber experiments as short circuit currents (I_sc_). The autocrine EP1 and EP4 pathways operate through G-proteins that indirectly stimulate sAC through increases in intracellular Ca^2+^ ([Ca^2+^]_i_), thereby amplifying the cAMP signal to increase CFTR conductance [21]. More prolonged exposures to H_2_O_2_ induces a decrease in resistance with concomitant increase in permeability. These changes are believed to represent alteration of epithelial barrier function. Thus, to better understand the mechanism underlying the H_2_O_2_-induced junctional disruption, we explored the role of the H_2_O_2_-mediated decreases in resistance and increases in permeability using primary NHBE cell cultures re-differentiated at the air liquid interface. These experiments showed that the H_2_O_2_-induced effects on resistance and permeability depended not only on direct inhibition of tyrosine protein phosphatases by H_2_O_2_, but also on a G-protein coupled receptor (GPCR) transduction path that involves the Ca^2+^-mediated stimulation of sAC activity and PKA.

## Methods

### Cell culture

Human airway epithelial cells were obtained from organ donors whose lungs were rejected for transplant. Consent was obtained through the Life Alliance Organ Recovery Agency of the University of Miami and the LifeCenter Northwest in WA according to IRB approved protocols. Epithelial cells from the lower trachea and bronchi were isolated as previously described [22, 23]. Air-liquid interface (ALI) cultures were allowed to differentiate for at least 2 weeks prior to experiments. All experiments were performed with date, passage and lung matched control cultures.

### Chemicals

DMEM, Ham’s nutrient F-12 and Hank’s balanced salt solution were purchased from Gibco, Life Technologies (Grand Island, NY). Gly-H 101 was from Calbiochem. Prostanoid receptor antagonists were from Cayman Chemicals. All other chemicals, unless otherwise stated, were purchased from Sigma Aldrich (St. Louis, MO).

### Ussing chambers

Snapwell filters containing differentiated NHBE cells were rinsed with Krebs-Henseleit (KH), and then mounted in Ussing chambers (EasyMount Chamber; Physiologic Instruments; San Diego, CA) with KH in apical and basolateral chambers. KH consisted of: 118 mM NaCl, 25 mM NaHCO_3_, 4.7 mM KCl, 1.2 mM MgSO_4_, 1.2 mM NaH_2_PO_4_, 1.2 mM CaCl_2_, 5.5 mM glucose, pH 7.35 when gassed with 95 % O_2_–5 % CO_2_. Solutions were maintained at 37 °C by heated water jackets and continuously bubbled with a 95 % O_2_–5 % CO_2_ mixture. To monitor I_SC_ and resistance, the transepithelial membrane potential was clamped at 0 mV with a 6-channel voltage clamp (model VCC MC6, Physiologic Instruments) using Ag/AgCl electrodes in agar bridges. Signals were digitized and recorded with DAQplot software (VVI Software, College Station, PA) via a LabJack A/D converter (LabJack Corp, Lakewood, CO). The input resistance of each filter was measured by application of 1 mV bipolar pulses of 2 s duration. After addition of amiloride, I_SC_ was allowed to stabilize and then H_2_O_2_ was included in the apical perfusate. Time scales were initialized at the time of mounting in the chamber. To explore the mechanisms responsible for this change in I_SC_, cultures were incubated apically and basolaterally with pharmacological agents for 20–50 min before and during apical exposure to H_2_O_2_.

### Permeability measurements

NHBE ALI cultures were preincubated for 10 min at 37 °C in KH-buffer (pH 7.35). ^14^C mannitol was added apically and initial apical to basolateral mannitol flux was determined [24]. Cells were washed apically and basolaterally three times to remove ^14^C mannitol, treated apically with H_2_O_2_ or inhibitors/activators, fresh ^14^C mannitol added apically and final mannitol flux was determined. Apparent permeability *P*_app_ was calculated for initial and final mannitol flux using the following equation: *P*_app_ = dQ/dT/A^.^C_0_ where dQ/dt is the flux determined from the amount transported (Q) over time (T) of the experiment, A is the surface area of the filter, and C_0_ is the initial concentration on the donor side. Results were expressed as mannitol *P*_app_ normalized to initial *P*_app_.

### Intracellular Ca^2+^ measurements

NHBE ALI cultures were loaded at room temperature for 2 h by gentle rocking of cultures in 10 μM fura-2 AM (ThermoFisher Scientific, Waltham, MA) in Dulbecco’s PBS containing 1 % glucose and 10 % FBS. Cultures were washed and mounted in a perfusion chamber in Krebs-Henseleit buffer at room temperature and ratiometric images captured and quantified as described previously [25].

### Immunocytochemistry

Fully differentiated NHBE cells in ALI cultures were fixed in 4 % paraformaldehyde in PBS, pH 7.4 for 15 min and permeabilized with 1 % Triton X-100 in PBS for 20 min at room temperature (RT). After permeabilization, cells were washed with PBS and then blocked with 3 % BSA in PBS for 1 h RT, followed by rabbit anti human EP1 receptor antibody (1 μg/ml, Cayman Chemical #101740 l) and mouse anti human acetylated α-tubulin (Sigma Cat. #T6793; 1:2000) in blocking solution and incubated overnight at 4 °C. Non-immune controls used normal rabbit IgG at 1 μg/ml. EP1 and acetylated α-tubulin were visualized with Alexa 488-labeled anti-rabbit antibody (4 μg/ml, Invitrogen) and Alexa 647 labeled anti-mouse antibody respectively. Nuclei were labeled with 4,6-diamidine-2-phenylindole (DAPI, KPL). Samples on membranes were mounted on slides with Fluoro-Gel (EMS; Cat. #17985-10) and fluorescent images were acquired on a Leica DM6000 microscope with a SP5 confocal module at the University of Miami McKnight Analytical Imaging Core Facility.

### Data analysis

The maximum change in I_SC_ following stimulation was normalized to baseline values obtained after addition of amiloride and other inhibitors. Replicate cultures from each lung donor and then all donors were averaged to give mean values. When multiple treatments were compared, changes were expressed as a fraction of date and lung matched controls. Mean values were compared by one-way ANOVA and if significant differences were obtained, by the Tukey Kramer HSD test. If means were not normal, the Wilcoxon or Kruskal-Wallis test was used (JMP software, SAS, Cary NC and Prism, Graphpad Software, La Jolla CA).

## Results

**Fig. 1:**
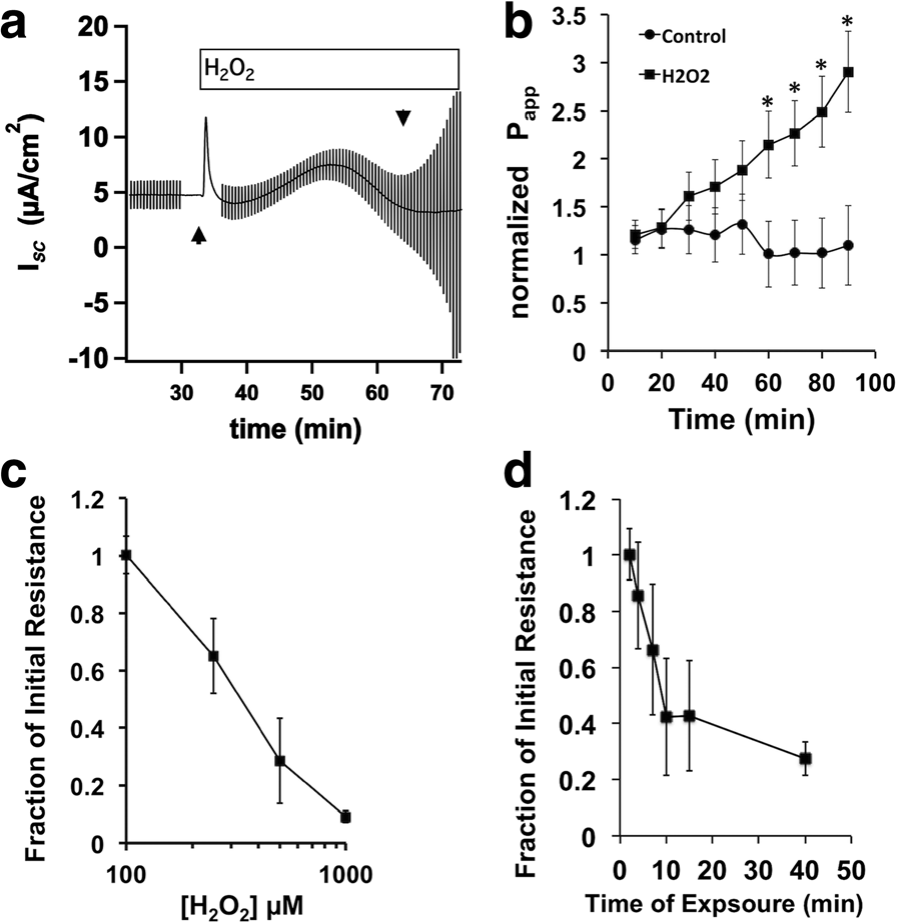
H_2_O_2_ effects on junctions of differentiated NHBE cells. NHBE cultures were mounted in Ussing chambers and exposed to H_2_O_2_ (panels **a**, **c**-**d**). Panel **a**. Acute exposure to apical 1 mM H_2_O_2_ elicits a transient increase in Isc (*upward arrow head*) and prolonged exposure induces a decrease in membrane resistance (*downward arrow head*). Note the size of the current after voltage pulses prior to the addition of H_2_O_2_ compared to the much larger current from pulses 40 min after exposure. Larger currents signify lower membrane resistances. For all experiments, transepithelial membrane resistances were measured 40–60 min after H_2_O_2_ exposure. Panel **b**. Prolonged exposure to apical 1 mM H_2_O_2_ elicits a concomitant increase in paracellular permeability to mannitol (circles: no treatment, squares: H_2_O_2_ treated, mean ± s.e.m., *n* = 7 cultures from 5 lung donors). Panel **c**. The observed decrease in membrane resistance after 40 min is dependent on the H_2_O_2_ concentration (mean ± s.e.m., *n* = 8 cultures from 4 donors). Panel **d**. The H_2_O_2_-induced change in resistance is dependent on the length of H_2_O_2_ exposure. Removing H_2_O_2_ from the perfusate within 5 min of exposure prevents the large change in resistance measured after 40 min

In Ussing chamber experiments, exogenously applied H_2_O_2_ changes membrane ion conductance and resistance in NHBE cells. Acutely, H_2_O_2_ leads to increases in CFTR-mediated conductance. Low H_2_O_2_ concentrations elicit sustained increases in I_sc_ that plateau above baseline [20, 21]. High H_2_O_2_ concentrations produce transient currents of larger amplitudes comprised of an increase to a peak followed by a decrease towards baseline (Fig. 1a, upward arrow head).

Prolonged apical exposures induced a large decrease in resistance (Fig. 1a, downward arrow head) that in some instances approached zero and a concomitant increase in paracellular permeability (Fig. 1b). Initial resistance values of differentiated NHBE cultures ranged from 200 to 1000 Ω/cm^2^. Apical H_2_O_2_ treatment decreased resistance as a function of starting resistance of the culture and not by a fixed absolute amount in each culture. Thus, resistance changes were expressed after normalization to the starting value for that culture at the beginning of the experiment. The decrease in membrane resistance depended on the H_2_O_2_ concentration (Fig. 1c), started to occur approximately 15 min after H_2_O_2_ exposure and lasted at least 1 h. The H_2_O_2_-induced change in resistance was observed even after exposure times as short as 10 min. Removal of H_2_O_2_ within 5 min of exposure, however, prevented the change in resistance (Fig. 1d).

Pre-treating NHBE cells apically with 1 mM H_2_O_2_ caused about a 50 % decrease in the forskolin-induced I_SC_ within 10 min of addition (control: 20.7 ± 3.6 μA, *n* = 9 lung donors vs H_2_O_2_-treated: 9.2 ± 2.1 μA, *n* = 9 lung donors, Additional file 1: Figure S1). Despite H_2_O_2_ having such a profound effect on resistance and CFTR activity, the effects were completely reversed within 24 h after treatment was stopped. Cultures exposed to apical 1 mM H_2_O_2_ for 1 h recovered normal resistance in agreement with studies using cell lines [10] and also recovered CFTR activity as reflected in the forskolin-stimulated I_SC_ returning to normal values when measured 24 h after H_2_O_2_ exposure (Additional file 1: Figure S2). Additionally, cultures continuously exposed to apical 1 mM H_2_O_2_ for 18 h also recovered normal resistance and forskolin-stimulated I_SC_ within 8 h after H_2_O_2_ removal.

**Fig. 2:**
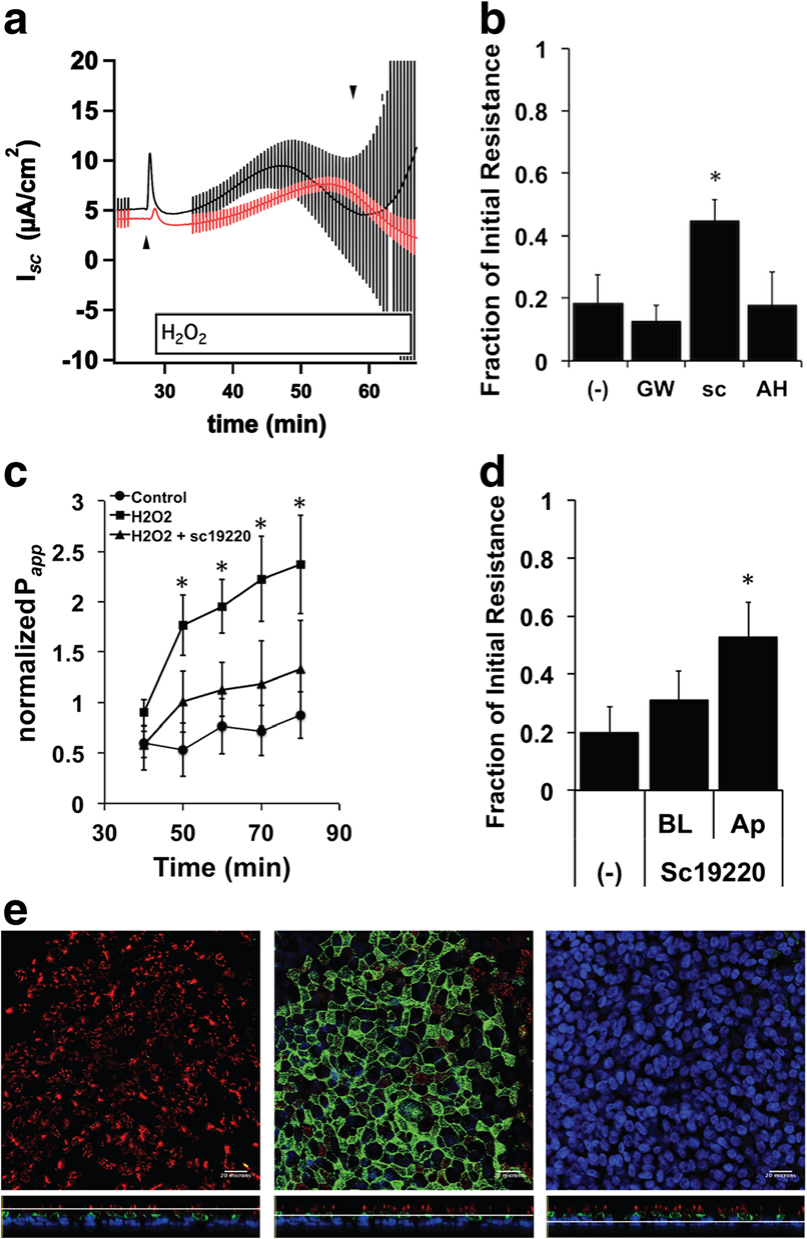
sc19220, an EP1 receptor antagonist, attenuates H_2_O_2_-associated changes in I_SC_ and transepithelial resistance and permeability. Panel **a**. Representative Ussing chamber experiment showing H_2_O_2_-induced I_SC_ with current pulses before and after H_2_O_2_ exposure. The EP1 receptor antagonist sc19220 (20 μM) not only diminishes H_2_O_2_-induced increases in I_SC_ (upward arrow head), but also rescues the decrease in membrane resistance associated with apical H_2_O_2_ exposure. Compare the pulse size of the sc19220 pretreated cells (red trace) with the much larger control pulses (black trace) after prolonged H_2_O_2_ exposure (downward arrow head). Panel **b**. Fully differentiated NHBE cells were mounted in Ussing chambers, treated with 10 μM amiloride followed by an EP1 or EP4 receptor antagonist in the apical and basolateral compartments for 20 min. The cells were then stimulated with H_2_O_2_ apically in the presence of current pulses to determine the membrane resistance before and after H_2_O_2_ exposure. Only the inhibition of the EP1 receptor with sc19220 (20 μM, *n* = 40 cultures from 26 donors) and not the inhibition of the EP4 receptor with GW627368X (200 nM, *n* = 6 cultures from 6 donors) or AH23848 (5 μM, *n* = 4 cultures from 4 donors, * = *p* < 0.05 compared to control), attenuates the decrease in resistance associated with H_2_O_2_ exposure. Panel **c**. Permeability of cultures treated with sc19220 and H_2_O_2_ were not significantly different to untreated control (*n* = 3 filters from 3 lungs, * = *p* < 0.05). Panel **d**. The protective effect of sc19220 on membrane resistance appeared to be an apical phenomenon, since only apical and not basolateral pre-treatment with the EP1 antagonist diminished the effects of H_2_O_2_ (*n* = 5 cultures from 5 donors, *p* < 0.05). Panel **e**, NHBE cultures were fixed and then stained with anti-EP1 receptor (green), acetylated α-tubulin (cilia, *red*) and DAPI (nuclei, *blue*) and then imaged by confocal microscopy. Confocal sections of combined color channels through the cilia (left), apical membrane (center) and nuclei (right) are shown with indications of the plane in the Z-stack below each panel. The images showed anti-EP1 receptor antibody (green) had an apical localization with a concentration near the apical junction between cells. Bars = 20 μm

H_2_O_2_ exposure leads to the production of prostaglandin compounds in NHBE cells and cell lines and results in stimulation of an EP1 and EP4-mediated, autocrine signaling pathway [19, 20]. In brain tissue [26] and intestinal epithelial cell lines [27, 28], EP1 receptor stimulation causes an increase in barrier permeability. Pre-treating redifferentiated NHBE cells with 20 μM sc19220, an EP1 receptor antagonist, for 20 min attenuated the decrease in membrane resistance and increase in permeability seen after exposure to apical 1 mM H_2_O_2_ (Fig. 2a–c). EP4 receptors were not involved in the H_2_O_2_-induced changes in resistance, since pretreatment for 20 min with EP4 receptor antagonists, GW627368X (200 nM) or AH23848 (5 μM), did not affect the H_2_O_2_-induced decrease in resistance (Fig. 2b). Also, application of sc19220 attenuated the decrease in membrane resistance when applied only to the apical compartment (Fig. 2d) and not to the basolateral compartment implicating EP1 receptors at the mucosal surface. Immunocytochemistry with anti-human EP1 antibody showed that EP1 receptors were localized to the apical portion of NHBE cells with the highest concentration at the apical cell border reminiscent of apical junctional complexes (Fig. 2e).

**Fig. 3:**
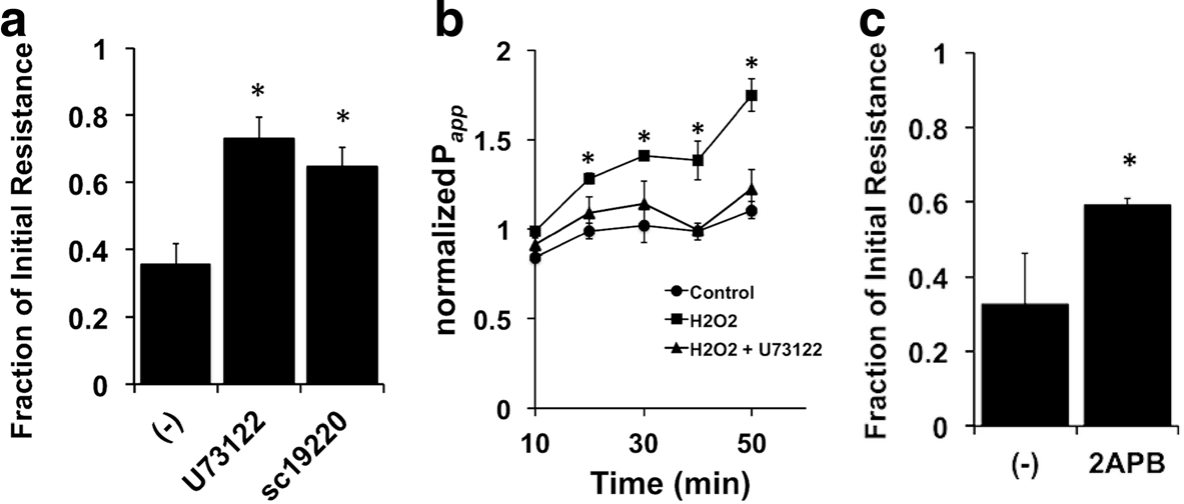
Phospholipase C is an integral part of the H_2_O_2_-induced changes in resistance and permeability. Panel **a**. Fully differentiated NHBE cells were mounted in Ussing chambers, treated with 10 μM amiloride and the phospholipase C inhibitor U73122 (20 μM) in the apical and basolateral compartments for 20 min. Cells were then stimulated with apical 1 mM H_2_O_2_ in the presence of current pulses to determine the membrane resistance before and after H_2_O_2_ exposure. Pre-treating NHBE cells with U73122 (*n* = 8 cultures from 8 donors, * = *p* < 0.05) attenuated the H_2_O_2_-induced decrease in membrane resistance in a similar fashion to that seen with the EP1 receptor antagonist sc19220 (*n* = 8 cultures from 8 donors, * = *p* < 0.05). Panel **b**. Parallel mannitol flux experiments also showed an attenuation of the concomitant increase in permeability associated with apical 1 mM H_2_O_2_ exposure (*n* = 4 cultures from 2 lung donors, * = *p* < 0.05 compared to control). Panel **c**. Inhibition of the IP_3_ receptor with 2-APB (200 μM) also attenuates H_2_O_2_-induced decreases in resistance, further supporting a role for [Ca^2+^]_i_ in the pathway (*n* = 3 cultures from 3 donors, * = *p* < 0.05)

It is known that EP1 receptors are coupled to Gq and that this complex activates phospholipase C. The observation that EP1 receptor inhibition attenuates the changes associated with H_2_O_2_ exposure suggested that the mechanistic pathway underlying the effect of H_2_O_2_ on membrane resistance and permeability operated at least in part through increases in [Ca^2+^]_i_. NHBE cells pre-treated for 20 min with the phospholipase C inhibitor U73122 (20 μM) showed an attenuation of the H_2_O_2_-induced decrease in resistance similar to that seen with the EP1 antagonist sc19220 (Fig. 3a). Parallel mannitol flux experiments also showed a decrease in H_2_O_2_-induced permeability changes when NHBE cells were pre-treated with U73122 (Fig. 3b). Inhibition of IP_3_ receptors with 2-aminoethoxydiphenyl borate (200 μM) also lead to a decrease in the H_2_O_2_-induced changes in resistance, further supporting a role of [Ca^2+^]_i_ in this pathway (Fig. 3c).

**Fig. 4:**
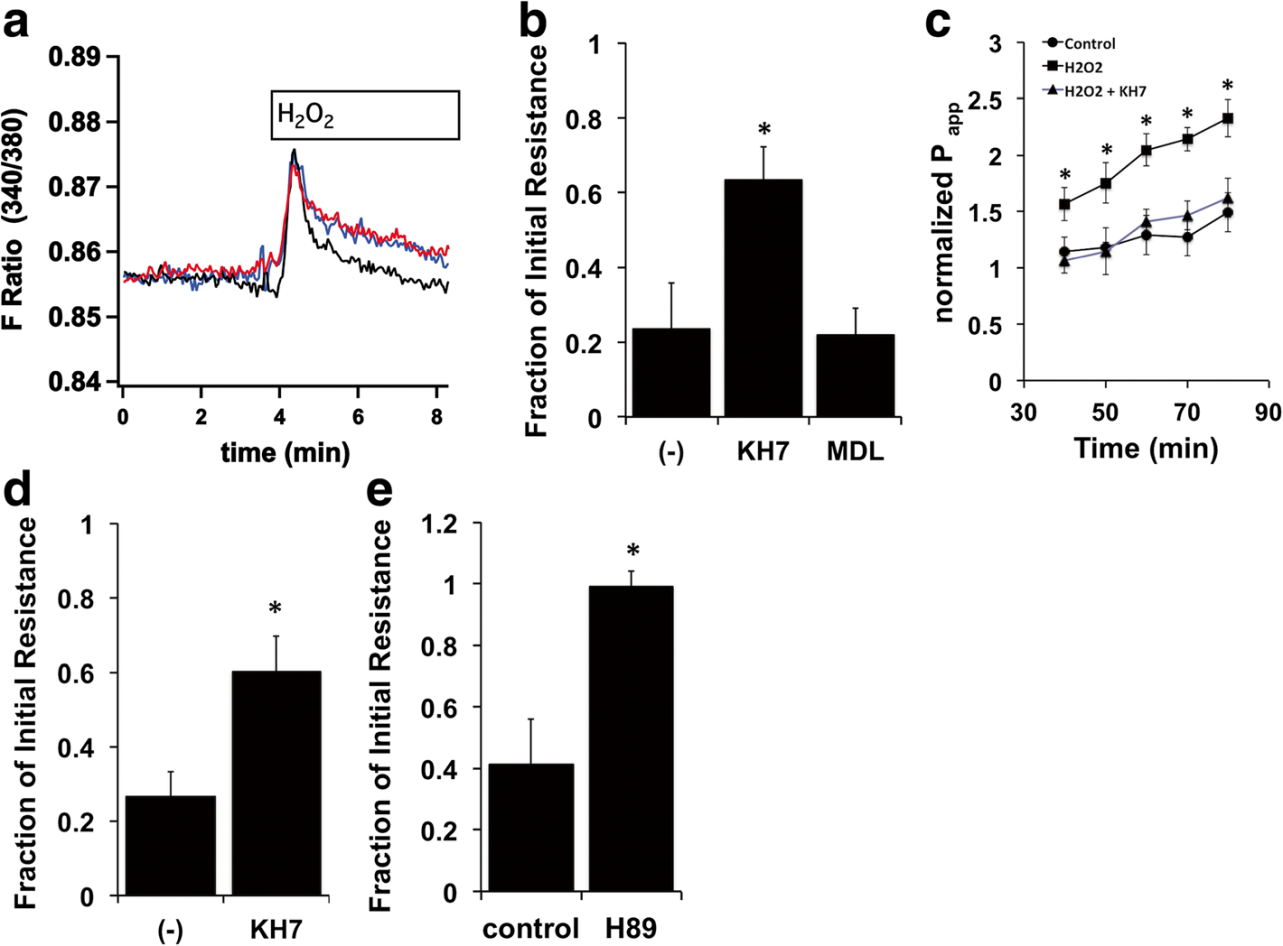
H_2_O_2_-induced changes in permeability and resistance are mediated by sAC. Panel **a**. Treatment with 400 μM H_2_O_2_ stimulated an increase in [Ca^2+^]_i_ in NHBE cells loaded with fura-2 AM (traces from three regions of interest). Panel **b**. sAC, but not tmAC, is involved in the H_2_O_2_-induced change in resistance. Fully differentiated NHBE cells were mounted in Ussing chambers, treated with 10 μM amiloride and the sAC inhibitor KH7 (25 μM) or the tmAC inhibitor MDL-12,330A (25 μM) in the apical and basolateral compartments for 20 min. The cells were then stimulated with apical 1 mM H_2_O_2_ in the presence of current pulses to determine the membrane resistance before and after H_2_O_2_ exposure. Pre-treating NHBE cells with KH7 attenuated the H_2_O_2_-induced decrease in membrane resistance while the tmAC inhibitor did not have an effect (*n* = 6 cultures from 6 donors, * = *p* < 0.05). Panel **c**. Inhibition of sAC attenuated the increase in permeability associated with prolonged H_2_O_2_ exposure (*n* = 4 lungs). Panel **d**. Lower concentrations of KH7 (10 μM) also had a significant effect on the H_2_O_2_-induced change in resistance (*n* = 6 cultures from 6 donors, * = *p* < 0.05). Panel **e**. NHBE cells were mounted in Ussing chambers and pretreated with 15 μM H89 for 20 min. The cells were then exposed to apical 1 mM H_2_O_2_ for 15 min. Inhibition of PKA attenuates the H_2_O_2_ induced decrease in resistance (*n* = 3 cultures from 3 donors, * = *p* < 0.05)

To confirm that H_2_O_2_-induced stimulation of phospholipase C via EP1 receptors led to an increase in [Ca^2+^]_i_, NHBE cells were loaded with fura-2 AM, mounted on the stage of a Nikon microscope and stimulated with 400 μM H_2_O_2_. H_2_O_2_ treatment resulted in an immediate increase in [Ca^2+^]_i_ (Fig. 4a). Since calcium is an activator of both sAC and tmAC, we pre-treated NHBE cells with either the sAC inhibitor KH7 (25 μM) or the tmAC inhibitor MDL-12,330A (25 μM) to determine a possible role of these enzymes in the H_2_O_2_-induced change in resistance or permeability. Inhibition of sAC attenuated the H_2_O_2_-induced decrease in resistance or increase in permeability (Fig. 4b–c). On the other hand, inhibition of tmAC did not significantly alter the change in resistance associated with H_2_O_2_ (Fig. 4b). Lower concentrations of KH7 (10 μM) also attenuated the H_2_O_2_-induced decrease in resistance significantly (Fig. 4d). Inhibition of PKA with H-89 (15 μM) led to a modest delay and attenuation of the H_2_O_2_-induced decrease in resistance. The involvement of PKA in the pathway became more evident when NHBE cells were washed free of H_2_O_2_ after a 15 min exposure (Fig. 4e). Inhibitors of CFTR, COX, PKC and EPAC did not alter the H_2_O_2_-induced change in resistance (data not shown).

**Fig. 5:**
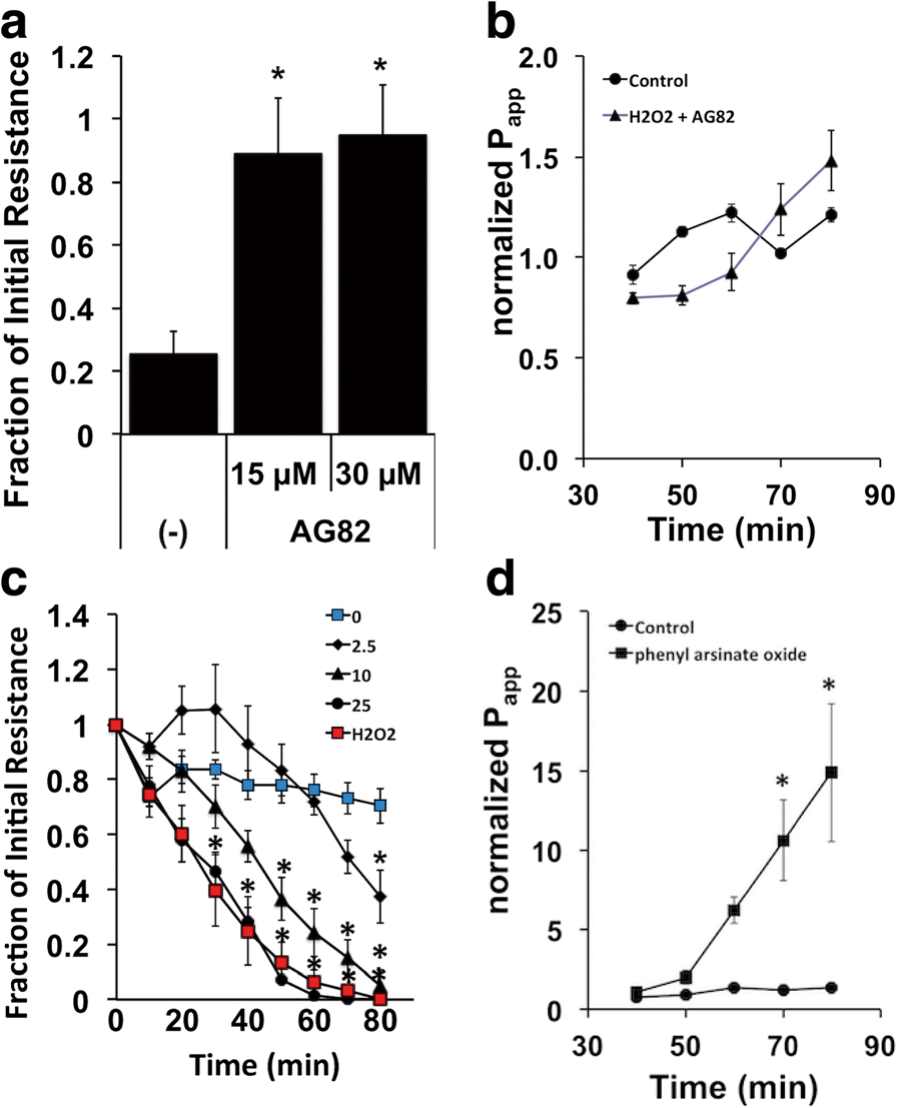
Phosphorylation and dephosphorylation regulates tight junction integrity. Panel **a**. Pre-treating NHBE cells with 15 or 30 μM AG82, a protein tyrosine kinase inhibitor, blocks the decrease in resistance associated with H_2_O_2_ exposure (mean ± s.e.m., *n* = 7 cultures from 6 lung donors each concentration, * = *p* < 0.05 compared to H_2_O_2_ alone). Panel **b**. Pretreatment of cultures with AG82 (15 μM) and H_2_O_2_ was not different from control untreated cultures: no increase in permeability seen (*n* = 3 cultures from 3 lungs). Panel **c**. The phosphatase inhibitor phenylarsine oxide decreased the resistance in NHBE cultures in a concentration dependent fashion (2.5–25 μM), similar to H_2_O_2_ (*n* = 4 cultures from 4 lung donors, * = *p* < 0.05 compared to no H_2_O_2_ control). Panel **d**. Mannitol flux experiments also showed an increase in permeability induced by phosphatase inhibition (25 μM phenylarsine oxide, *n* = 3 cultures from 3 lungs, * = *p* < 0.05)

Maintenance of junctional integrity requires a balanced interplay between protein phosphorylation by kinases and dephosphorylation by phosphatases (e.g., [29]). This was evident when NHBE cells are treated with either a protein tyrosine kinase or phosphatase inhibitor. Pretreating NHBE cells with 15 μM or 30 μM AG82, a protein tyrosine kinase inhibitor, greatly inhibited the decrease in resistance and increase in permeability after H_2_O_2_ exposure (Fig. 5a–b). On the other hand, treating cells with phenylarsine oxide, a protein tyrosine phosphatase inhibitor, elicited a concentration-dependent decrease in resistance and increase in permeability similar to that seen with H_2_O_2_ (Fig. 5c–d). Thus, it appeared that both PKA and a tyrosine kinase were involved in mediating H_2_O_2_-induced changes in barrier function.

## Discussion

Most studies of H_2_O_2_ effects on junctions have used epithelial cell lines to demonstrate that H_2_O_2_ compromises barrier function as measured by increased paracellular permeability and decreased transepithelial resistance. These studies on cell lines show that the pathway initiated by H_2_O_2_ is multifactorial and cell type specific [8]. Thus, the mechanisms are not clear, especially not in primary cells or tissues. This study uses fully differentiated primary epithelia in vitro. The data suggested that the H_2_O_2_ effect on bronchial epithelial junctions involves, in part, a specific PKA pathway, initiated by EP1 receptor stimulation, leading to Ca^2+^ activation of sAC and PKA, which in turn mediated changes in the function of apical junctions. In NHBE, the H_2_O_2_-activated PKA pathway was seen along with H_2_O_2_ effects on activation of tyrosine kinases [30] and inactivation of tyrosine phosphatases [31] both known to interfere with junctions.

Previous studies have shown that H_2_O_2_ -mediated decreases in resistance and increases in permeability occur through a variety of changes in protein phosphorylation, including inhibition of tyrosine phosphatases (e.g., [32]), increased tyrosine kinase activity (e.g., [29]) and increased serine/threonine dephosphorylation [15]. In NHBE cells, direct treatment with the phosphatase inhibitor phenylarsine oxide induced a change in resistance similar to H_2_O_2_, and pre-treatment with the protein tyrosine kinase inhibitor AG82 caused the H_2_O_2_-induced decrease in resistance to be greatly attenuated. Treatment of NHBE cells with H_2_O_2_ caused, in addition to changes in protein tyrosine phosphorylation, stimulation of PKA that also led to reduced resistance and higher permeability. Interestingly, H_2_O_2_-mediated changes in resistance could only be blocked by PKA inhibition if H_2_O_2_ exposure was short (15 min), suggesting longer treatments activated a second parallel, PKA-independent pathway to alter intercellular junctions. Nevertheless, activation of sAC appeared absolutely necessary for H_2_O_2_-mediated changes in junctions since a sAC inhibitor blocked changes induced by both short and long exposures. These data suggest that sAC may be compartmentalized in a place allowing it to solely be responsible. sAC has been shown to be localized at the ciliated, apical membrane of NHBE and in cilia [33, 34].

Acute application of H_2_O_2_ to normal human airway epithelial cells induces transient increases in CFTR conductance via a prostanoid signaling pathway involving increased release of prostaglandin E2, activation of prostaglandin E receptors, EP1 and EP4 [20, 21] that signal via Gq and Gs, respectively. The data here showed that EP1 but not EP4 signaling was a key aspect of H_2_O_2_-induced changes in transepithelial resistance and paracellular permeability. Inhibition of PLC and IP_3_ receptors caused an attenuation of the H_2_O_2_-induced decrease in resistance and increase in permeability. However, blocking the EP1 signaling pathway was not sufficient to completely inhibit H_2_O_2_ mediated junctional changes, supporting the idea that more than one pathway worked in parallel to induce changes in resistance and permeability. Since sAC is activated by increases in [Ca^2+^]_i_, the data were consistent with a pathway that involves H_2_O_2_ induction of prostaglandin release [20, 21], activation of EP1 receptors to increase [Ca^2+^]_i_ and activate sAC to produce cAMP for PKA activity. Whether this pathway interacts with, or modulates tyrosine phosphorylation that also regulates junctions is not known.

Inflammatory airway diseases are often characterized by defective barrier function and coincidentally are associated with elevated H_2_O_2_ presumably derived from NADPH oxidases of both infiltrating phagocytes as well as epithelial cells. The knowledge that H_2_O_2_ contributes to barrier disruption suggests a possible mechanistic link between H_2_O_2_ and tight junction changes in inflammation. The data presented here implicate EP1-mediated activation of sAC to increase epithelial cAMP and increase barrier disruption. In contrast, modulators that increase cAMP, relax airway smooth muscle and are used to treat asthma and COPD might also lead to counterproductive cAMP increases in epithelial cells. Thus, the EP1 signaling pathway or sAC itself might be possible therapeutic targets to prevent deleterious changes in epithelia barrier function.

## Conclusion

H_2_O_2_-induced changes in NHBE barrier function occur through an autocrine prostanoid signaling pathway that increased [Ca^2+^]_i_, activated sAC and altered epithelial resistance and permeability. Previous studies in brain and intestinal cells have shown prostaglandin-induced increases in permeability [26–28], suggesting a similar sAC-mediated mechanism of action of H_2_O_2_ on epithelial barrier function may occur in a variety of tissues.

## Additional file

Additional file 1: Figure S1. H_2_O_2_ inhibition of forskolin-stimulated I_S__C_. NHBE cultures were mounted in Ussing chambers and treated with either 10 μM forskolin or 1mM H_2_O_2 _followed by 10 μM forskolin. Panel a, representative traces of forskolin-induced CFTR activity with (red trace) and without (black trace) H_2_O_2_ pre-treatment. Panel b, I_SC_ values with (treated) and without (control) H_2_O_2_ pre-treatment (mean ± s.e.m., n = 9 lung donors). H_2_O_2_ treatment reduced the forskolin-stimulated I_SC_ by approximately 50 %. **Figure S2.** Recovery of resistance and forskolin-stimulated I_SC_ after H_2_O_2_ treatment. NHBE cultures were exposed to apical 1 mM H_2_O_2_ for 1h. After removal of H_2_O_2_ the cultures were returned to the CO_2_ incubator for 23h and then placed in Ussing chambers for measurement of both resistance and forskolin-stimulated I_S__C_ (time point 24h). Panel a, I_SC_ before and 23h after treatment are shown (mean ± s.e.m., n = 3 lung donors). Forskolin-stimulated I_SC_ currents returned to normal within 23h after treatment. Panel b, Epithelial resistance before and 23h after treatment are shown (mean ± s.e.m., n = 3 lung donors). Resistance returns to normal within 23h after treatment. (DOCX 633 kb)

## Abbreviations

[Ca^2+^]_i_: intracellular calcium concentration
ALI: air liquid interface
GPCR: G-protein coupled receptor
I_sc_: short circuit current
KH: Krebs- Henseleit buffer
NHBE: normal human bronchial epithelial
sAC: soluble adenylyl cyclase
tmAC: transmembrane adenylyl cyclase
