# Supplementary material for: Soluble adenylyl cyclase mediates hydrogen peroxide-induced changes in epithelial barrier function
Source: Respir Res. 2016 Feb 8;17:15. doi: 10.1186/s12931-016-0329-4 (PMC4746823; doi:10.1186/s12931-016-0329-4)
Supplement: Additional file 1: Figure S1. — H2O2 inhibition of forskolin-stimulated IS C. NHBE cultures were mounted in Ussing chambers and treated with either 10 μM forskolin or 1mM H2O2 followed by 10 μM forskolin. Panel a, representative traces of forskolin-induced CFTR activity with (red trace) and without (black trace) H2O2 pre-treatment. Panel b, ISC values with (treated) and without (control) H2O2 pre-treatment (mean ± s.e.m., n = 9 lung donors). H2O2 treatment reduced the forskolin-stimulated ISC by approximately 50 %. Figure S2. Recovery of resistance and forskolin-stimulated ISC after H2O2 treatment. NHBE cultures were exposed to apical 1 mM H2O2 for 1h. After removal of H2O2 the cultures were returned to the CO2 incubator for 23h and then placed in Ussing chambers for measurement of both resistance and forskolin-stimulated IS C (time point 24h). Panel a, ISC before and 23h after treatment are shown (mean ± s.e.m., n = 3 lung donors). Forskolin-stimulated ISC currents returned to normal within 23h after treatment. Panel b, Epithelial resistance before and 23h after treatment are shown (mean ± s.e.m., n = 3 lung donors). Resistance returns to normal within 23h after treatment. (DOCX 633 kb) [file 12931_2016_329_MOESM1_ESM.docx]

Additional file 1

Soluble adenylyl cyclase mediates hydrogen peroxide-induced changes in epithelial barrier function

Pedro Ivonnet^1^, Hoshang Unwalla^2^, Matthias Salathe^1^ and Gregory E. Conner^3^*

Figure S1


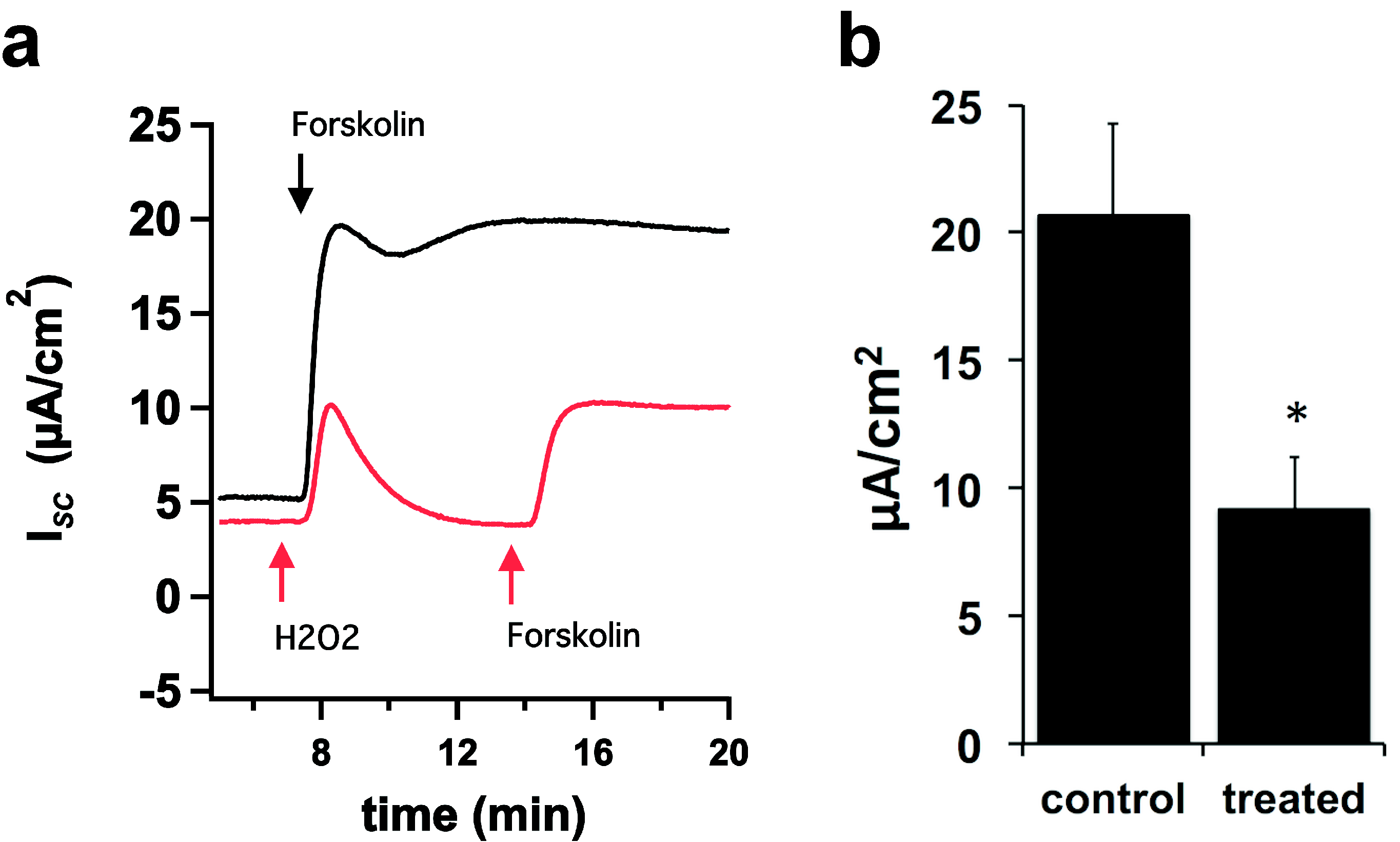


H_2_O_2_ inhibition of forskolin-stimulated I_SC_. NHBE cultures were mounted in Ussing chambers and treated with either 10 µM forskolin or 1mM H_2_O_2_ followed by 10 µM forskolin. Panel a, representative traces of forskolin-induced CFTR activity with (red trace) and without (black trace) H_2_O_2_ pre-treatment. Panel b, I_SC_ values with (treated) and without (control) H_2_O_2_ pre-treatment (mean ± s.e.m., n = 9 lung donors). H_2_O_2_ treatment reduced the forskolin-stimulated I_SC_ by approximately 50%.

Figure S2


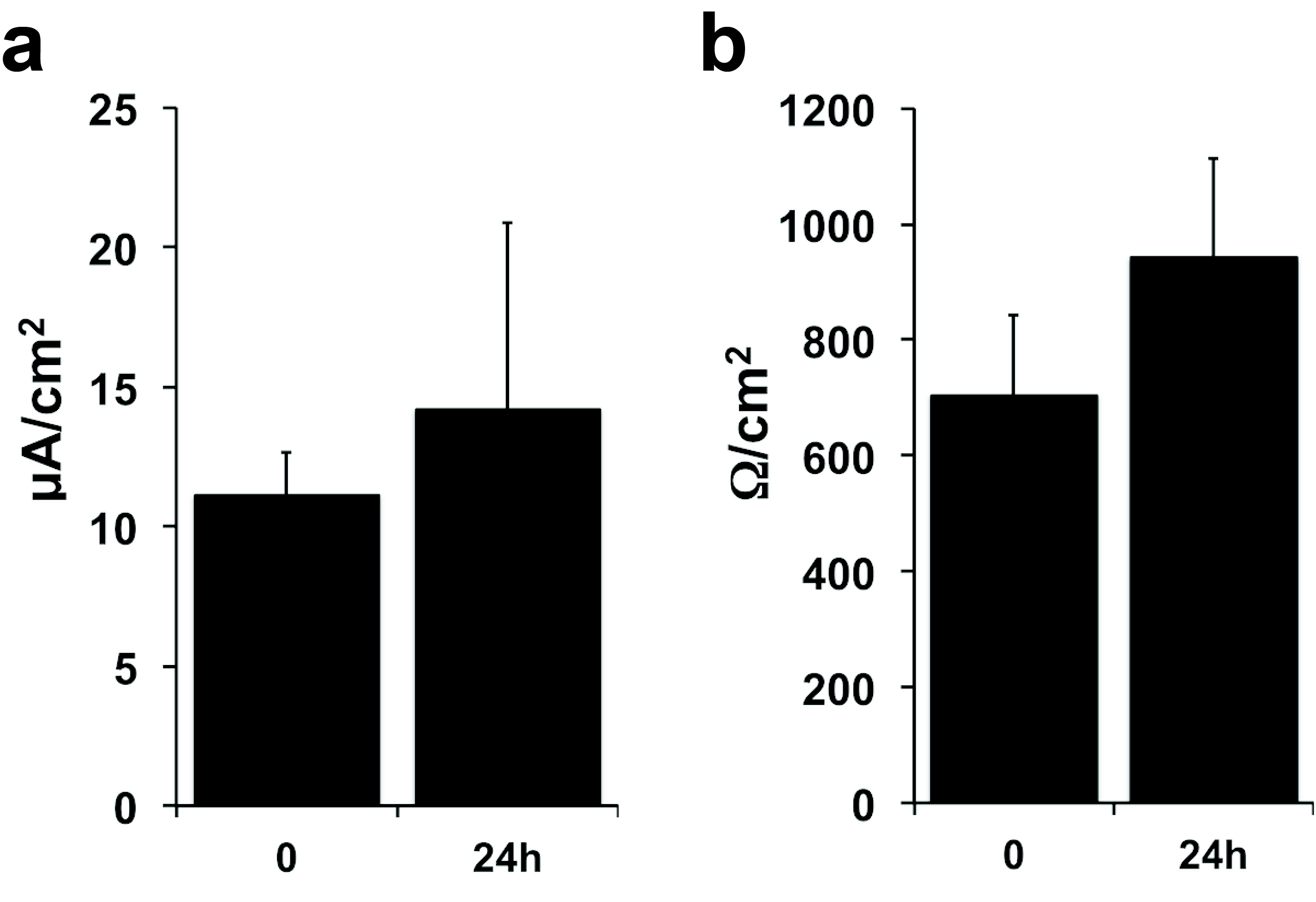


Recovery of resistance and forskolin-stimulated I_SC_ after H_2_O_2_ treatment. NHBE cultures were exposed to apical 1mM H_2_O_2_ for 1h. After removal of H_2_O_2_ the cultures were returned to the CO_2_ incubator for 23h and then placed in Ussing chambers for measurement of both resistance and forskolin-stimulated I_SC_ (time point 24h). Panel a, I_SC_ before and 23h after treatment are shown (mean ± s.e.m., n = 3 lung donors). Forskolin-stimulated I_SC_ currents returned to normal within 23h after treatment. Panel b, Epithelial resistance before and 23h after treatment are shown (mean ± s.e.m., n = 3 lung donors). Resistance returns to normal within 23h after treatment.
